# The Prostate-Associated Gene 4 (PAGE4) Could Play a Role in the Development of Benign Prostatic Hyperplasia under Oxidative Stress

**DOI:** 10.1155/2022/7041739

**Published:** 2022-05-19

**Authors:** Yan Li, Jianmin Liu, Daoquan Liu, Zhen Wang, Yongying Zhou, Shu Yang, Feng Guo, Liang Yang, Xinhua Zhang

**Affiliations:** Department of Urology, Zhongnan Hospital of Wuhan University, Wuhan, China

## Abstract

Benign prostatic hyperplasia (BPH) is a common disease in elderly men with uncertain molecular mechanism, and oxidative stress (OS) has also been found associated with BPH development. Recently, we found that prostate-associated gene 4 (PAGE4) was one of the most significantly changed differentially expressed genes (DEGs) in BPH, which can protect cells against stress stimulation. However, the exact role of PAGE4 in BPH remains unclear. This study is aimed at exploring the effect of PAGE4 in BPH under OS. Human prostate tissues and cultured WPMY-1 and PrPF cells were utilized. The expression and localization of PAGE4 were determined with qRT-PCR, Western blotting, and immunofluorescence staining. OS cell models induced with H_2_O_2_ were treated with PAGE4 silencing or PAGE4 overexpression or inhibitor (N-acetyl-L-cysteine (NAC)) of OS. The proliferation activity, apoptosis, OS markers, and MAPK signaling pathways were detected by CCK-8 assay, flow cytometry analysis, and Western blotting. PAGE4 was shown to be upregulated in human hyperplastic prostate and mainly located in the stroma. Acute OS induced with H_2_O_2_ increased PAGE4 expression (which was prevented by OS inhibitor), apoptosis, cell cycle arrest, and reactive oxygen species (ROS) accumulation in WPMY-1 and PrPF cells. siPAGE4 plus H_2_O_2_ potentiated H_2_O_2_ effect via reducing the p-ERK1/2 level and increasing p-JNK1/2 level. Consistently, overexpression of PAGE4 offset the effect of H_2_O_2_ and partially reversed the PAGE4 silencing effect. However, knocking down and overexpression of PAGE4 alone determined no significant effects. Our novel data demonstrated that augmented PAGE4 promotes cell survival by activating p-ERK1/2 and decreases cell apoptosis by inhibiting p-JNK1/2 under the OS, which could contribute to the development of BPH.

## 1. Introduction

Benign prostatic hyperplasia (BPH) refers to the nonmalignant growth of prostate tissue, and the incidence of this disease increases with age [1, 2]. In fact, the histological prevalence of BPH is 50% to 60% in men at 60 years of age and 80% to 90% in men over 70 years of age [3]. The secondary lower urinary tract symptoms (LUTS) impair the quality of their life [4]. Histopathologically, it results from a loss of homeostasis between cell proliferation and cell death with cell proliferation being dominant [5]. In addition to androgen and age dependence, androgen-estrogen ratio, the interaction between stromal and epithelial cells, and inflammation, as well as growth factors, are the other accepted predisposing BPH factors [6–8]. However, the exact pathophysiology of these mechanisms remains unclear.

In the past decades, oxidative stress (OS) has been found associated with BPH development, progression, and the response to therapy [9]. OS is defined as the imbalance between the production and scavenging of reactive oxygen species (ROS) [10]. It is thought that OS could produce oxidative DNA damage, defective DNA repair, and genomic instability, leading to an increase in mutations, some of which may promote cell transformation and accelerate proliferation [11].

Genomics technologies have led to deeper understanding of many human diseases. More than 15 years ago, DNA microarrays were used to explore BPH [12]. Recently, our group had performed mRNA expression profiling of three normal human prostates and five BPH tissues, and our microarray data (serial number: GSE119195) identified a total of 198 differentially expressed genes (DEGs) [13, 14]. Among these DEGs, prostate-associated gene 4 (PAGE4) was one of the genes significantly upregulated in BPH tissues. Although PAGE4 is expressed at basal levels in certain fetal and adult reproductive organs such as placenta and uterus [15], PAGE4 is clearly prostate-specific in men. In addition, its expression is highly dynamic. PAGE4 protein is highly upregulated in both fetal prostate epithelium and stromal cells at 21 weeks of gestational age [16], while it is almost undetectable in normal adult prostate [17]. Accumulated evidences have shown that PAGE4 is reexpressed in pathological human prostate tissues, especially in symptomatic benign prostatic hyperplasia [18], as well as prostate cancer [19–21]. PAGE4, as a kind of intrinsically disordered protein, can protect cells against stress stimulation [19]. In the cellular process, it has been found that PAGE4 is mainly located in mitochondria, which are the main site of oxidative stress. Indeed, studies found that PAGE4 was upregulated when prostate cancer cells were exposed to various stress inducers (including inflammatory stress and oxidative stress) [22]. However, how PAGE4 influences BPH under the condition of oxidative stress is still unclear.

In our current study, the relative expression of PAGE4 at transcriptional and translational levels was analyzed in human BPH prostates and human normal prostates. Furthermore, two cell models of PAGE4 deficiency or upregulation were established to investigate the functional activities of PAGE4. Also, the underlying mechanisms of PAGE4 protection of cells from OS were focused in the study.

## 2. Materials and Methods

### 2.1. Human Prostate Tissue Samples

Human hyperplastic prostate tissues were obtained from ten male patients (mean age, 68.3 ± 3.5 years) who underwent transurethral resection of the prostate in the Department of Urology, Zhongnan Hospital of Wuhan University. All samples exhibited BPH without tumor infiltration and intraepithelial neoplasia, which were confirmed by two separate pathologists. Normal prostate tissue was obtained from ten young brain-dead men (mean age, 31.7 ± 2.5 years) undergoing donation in the Organ Transplant Center of Zhongnan Hospital, with pathological exam showing no hyperplasia. Prostate tissues were divided into two strips and were, respectively, stored in liquid nitrogen for PCR and Western blotting analyses and in 10% neutral buffered formalin for immunofluorescence microscopy. All human samples were obtained after the approval of the Hospital Human Investigation Committee and receiving the written informed consent of all patients or their relatives. All human research was carried out in accordance with the principles of the Helsinki Declaration. The study was approved by the Ethics Committee of Zhongnan Hospital of Wuhan University, and samples were collected and processed in accordance with the approved guidelines.

### 2.2. Cell Culture and H_2_O_2_ Treatment

SV40 large-T antigen-immortalized stromal cell line WPMY-1 (Cat. #GNHu36) was purchased from the Stem Cell Bank, Chinese Academy of Sciences in Shanghai, China. Cell lines were identified at the China Center for Type Culture Collection in Wuhan, China. Primary prostate fibroblast (PrPF) was purchased from the Procell Co., Ltd. In Wuhan, China.WPMY-1 cells were cultured in DMEM medium (Gibco, China) containing 1% penicillin G sodium/streptomycin sulfate and 5% fetal bovine serum (FBS; Gibco, Australia). Fibroblasts were cultured in a DMEM : F12 1 : 1 (Thermo Fisher Scientific, MA, USA) mixture supplemented with 10% FBS (Thermo Fisher Scientific, MA, USA), 1% penicillin–streptomycin, 1 *μ*g/ml EGF (PeproTech, NJ, USA), and 1 *μ*g/ml bFGF (PeproTech, NJ, USA). All the cell lines were cultured in a humidified atmosphere consisting of 95% air and 5% CO_2_ at 37°C. Medium required by different cells were utilized to dilute 3% H_2_O_2_ (BD Biosciences, USA) into concentrations of 100 *μ*M, 200 *μ*M, 400 *μ*M, and 800 *μ*M for subsequent experiments. To stimulate oxidative stress *in vitro*, medium containing 400 *μ*M H_2_O_2_ was incubated with the cells for 8 h prior to analysis. 5 mM N-acetyl-L-cysteine (NAC, Sigma-Aldrich) was given to cells for 8 h before collection.

### 2.3. CCK-8 Assay

Cell Counting Kit-8 (MedChemExpress, China) was used to determine cell proliferation activity. After cell treatment, CCK-8 working solution of 10% volume fraction was added to the cells and cultured in a 37°C incubator for 4 h. The readings were recorded by a microplate reader (cat. no. SpectraMaxM2; Molecular Devices, Sunnyvale, CA, USA), and the absorbance at 450 nm was measured at the same time for each day.

### 2.4. Total RNA Extraction, Reverse Transcription, and Quantitative Real-Time PCR (qRT-PCR)

Total RNA was extracted from prostate tissues and cells using the RNeasy Mini Kit (Qiagen, Germany) according to the instructions and a centrifuge (Eppendorf, USA) combined with QIAshredder (Qiagen, Germany) to increase the quantity and quality of total RNA isolated. Each RNA sample was digested with DNase I (RNase-Free DNase Set, Qiagen, Germany) to remove contamination of the genomic DNA. The quantity of isolated RNA was detected with a NanoDrop® ND-1000 UV-Vis spectrophotometer (Thermo Scientific, USA). 1 *μ*g of total RNA isolated from prostate tissues or prostate cells was mixed with oligo (dT) 12–18 primers to synthesize first-strand cDNA by using Revert Aid First Strand cDNA Synthesis Kit (Thermo Scientific, China). 1 *μ*g cDNA was used for each individual polymerase chain reaction (PCR) reaction in a final volume of 20 *μ*l. All primers to be used with the SYBR Premix Ex Taq II (Takara Bio, China) were tested for optimal annealing temperatures, and PCR conditions were optimized with gradient PCRs on a Bio-Rad (Hercules, CA, USA) CFX96 system. The 2^-*ΔΔ*ct^ method was used to calculate the relative changes in expression. Primer sequences and annealing temperatures are summarized in Table 1.

### 2.5. Knockdown and Overexpression of PAGE4 in the Prostate Cells

PAGE4-targeted specific small interfering RNA (siRNA) was synthesized by GenePharma Ltd., in Shanghai, China. According to the instructions, WPMY-1 and PrPF were transfected with PAGE4-siRNA (SI-PAGE4) using Lipofectamine 2000 (Invitrogen, USA). The sense sequences of si-PAGE4 were as follows: si-1, 5′-GGAACCACCAACUGACAAUTTAUUGUCAGUUGGUGGUUCCTT-3′; si-2, 5′-GAAGGUGAUUGCCAGGAAATTUUUCCUGGCAAUCACCUUCTT-3′; si-3, 5′-CCUAGGAAAUUGACACUAUTTAUAGUGUCAAUUUCCUAGGTT-3′. And the sense sequence of si-control was 5′-UUCUCCGAACGUGUCAGUGACAUUAAGAUUCAGGGUTT-3′. After transfection by siPAGE4 for 48 h, alterations of PAGE4 at transcriptional and protein levels were evaluated by the qRT-PCR and Western blot.

PAGE4 cDNA was polymerase chain reaction (PCR) amplified from a cDNA library of human prostate cell lines and then cloned into a 2x FlagpcDNA3 empty vector performed with a one-step method to construct the homologous recombination vectors. After transfection by plasmid for 48 h, alterations of PAGE4 at transcriptional and protein levels were evaluated by qRT-PCR and Western blot.

### 2.6. Western Blot Analyses

The prostate cells were sonicated and lysed in RIPA buffer containing protease inhibitor and phosphatase inhibitor (Sigma-Aldrich, USA) on ice for 30 min and then centrifuged at 12,000 *g* for 15 min to collect supernatant. The concentrations of protein were determined by Bradford protein assay (Bio-Rad, Germany) using bovine serum albumin (BSA) as standard. The isolated total protein was resolved using 6–15% SDS-PAGE and transferred to PVDF membrane (Millipore, USA). Membranes were blocked by 5% nonfat milk and incubated with primary antibodies (Table 2) at 4°C for overnight. After washing, the membranes were incubated with secondary antibody (Table 3) at room temperature for 2 h. Bands were visualized using an enhanced chemiluminescence (ECL) kit (Thermo Scientific Fisher, Waltham, MA, USA) and detected by Kodak Biomax MR films.

### 2.7. Flow Cytometry Analysis

For cell cycle analysis, 1 × 10^6^ cells were harvested and fixed in 70% ice-cold ethanol at –20°C overnight. After centrifugation, pellets were resuspended with PBS containing 50 *μ*g/ml propidium iodide (Sigma-Aldrich, USA) and 0.1 mg/ml RNase A (20 *μ*g/ml in PBS) in the dark. After incubation at 37°C for 30 min, the DNA content distribution was analyzed by flow cytometry analysis (Beckman, Cat. #FC500). For apoptosis analysis, after transfection for 48 h, cells were collected, washed with PBS, and stained with FITC Annexin V Apoptosis Detection Kit I (BD Biosciences, USA) and analyzed by the flow cytometry analysis.

### 2.8. ROS Detection by Staining with DCFH-DA

The fluorescent probe 2′,7′-dichlorofluorescin diacetate (DCFH-DA Sigma-Aldrich, USA) was used to evaluate intracellular ROS levels. After transfection of cells and growth for 48 h, 10 *μ*mol of DCFH-DA (Sigma, USA) was added to 1 ml medium and incubated at 37°C for 30 min. Thereafter, the cells were washed three times with PBS and submitted to flow cytometry analysis.

### 2.9. Immunofluorescence Staining for Human Prostate Tissues

Frozen normal and BPH human prostate tissues were sectioned in 10 *μ*m thick slices and thawed, mounted onto glass slides using a cryostat (Leica CM 1850, Wetzlar, Germany), air-dried, and fixed for 10 min in ice-cold acetone. Slides were washed in PBS and incubated for 2 h in a mixture of PBS supplemented with 0.2% Triton X-100 and 0.1% bovine serum albumin. After incubation overnight with the primary antibody mixture of *α*-SMA and PAGE4 antibody (Table 2), the secondary antibodies (Jackson ImmunoResearch Inc.) labelled with FITC-conjugated anti-mouse IgG (1 : 200) and Cy3-conjugated anti-rabbit IgG (1 : 1000) were used to visualize the localization of the two primary antibodies. DAPI (Table 3) was used for staining the nucleus. Visualization was done with a Laser Scanning Confocal Microscope (Olympus, Tokyo, Japan).

### 2.10. Immunofluorescence Staining for Human Prostate Cells

For cell immunofluorescence microscopy, WPMY-1 and PrPF cells were cultured as aforementioned, followed by seeding on 12 mm coverslips and washing by ice-cold phosphate-buffered saline (PBS, pH = 7.4). The coverslips were then fixed with 4% paraformaldehyde (PFA) for 30 min, followed by 0.1% Triton X-100 incubation and blocked in goat serum for 30 min at room temperature and then incubated with primary antibody (Table 2) at room temperature for 2 h, washed with PBS, and incubated with Cy3-labeled or FITC-labeled secondary antibody (Table 3) for 1 h. Nuclei were labeled with DAPI (2 *μ*g/ml). Visualization was done with a Laser Scanning Confocal Microscope (Olympus, Tokyo, Japan).

### 2.11. Statistical Analysis

All analyses were performed at least three times and represented data from three individual experiments. The data values are expressed as the means ± standard deviation (SD). Statistical analysis was performed using Student's *t*-test (two groups compared) and one-way ANOVA (multiple means compared) with SPSS 22.0. Statistical significance was considered as a *p* value < 0.05.

## 3. Results

### 3.1. The Expression and Localization of PAGE4 in Human Prostate Tissues

**Figure 1:**
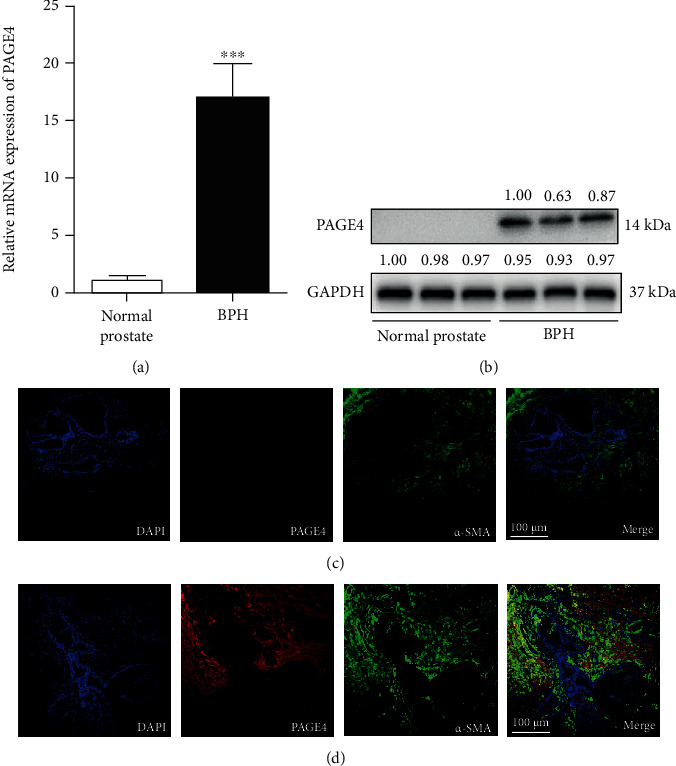
The expression of PAGE4 in prostate. (a) qRT-PCR analysis demonstrating the expression level of PAGE4 gene in BPH tissue (*n* = 10) and normal prostate tissue (*n* = 10) (b) Immunoblot assay revealed the protein expression of PAGE4 in normal prostate tissues and BPH tissues. Immunolocalization of PAGE4 for (c) normal prostate tissues and (d) BPH tissues. DAPI (blue) shows nuclear staining; Cy3-immunofluorescence (red) shows PAGE4 protein; *α*-SMA (green) indicates stromal staining. Sections of all samples were used for immunofluorescence experiments, and representative graphs were selected for figures. GAPDH is used as loading control. ^∗∗∗^: *p* < 0.001. Student's *t*-test. The scale bars are 100 *μ*m.

For human BPH and normal prostate samples, *n* = 10 each. In hyperplastic prostate, the mRNA level of PAGE4 was significantly increased over fifteenfold (Figure 1(a)), and the protein level of PAGE4 revealed significant upregulation, which could almost not be detected in normal prostate tissue (Figure 1(b)). Consistently, immunofluorescence staining demonstrated that PAGE4 was barely detectable in normal prostate tissue (Figure 1(c)) but it was richly expressed in hyperplastic tissues. Moreover, PAGE4 was mainly localized in the stroma (Figure 1(d)). Therefore, WPMY-1 cell line and PrPF were chosen for subsequent experiments.

### 3.2. Expression Level of PAGE4 Was Increased under ROS Stimulation in Prostate Stromal Cells

**Figure 2:**
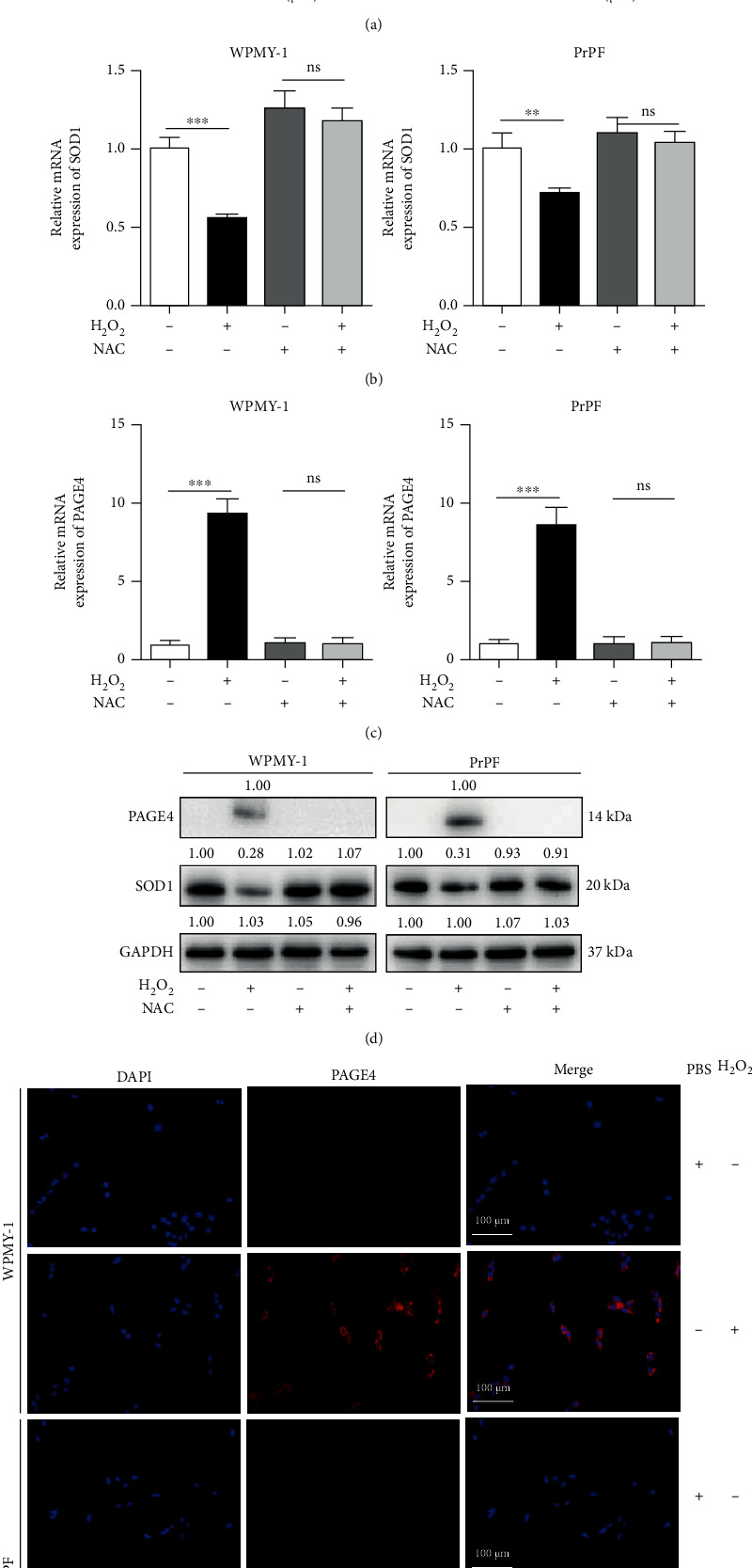
Expression of PAGE4 when exposed to H_2_O_2_ in WPMY-1 and PrPF cells. (a) The blank control group and 100, 200, 400, and 800 *μ*M H_2_O_2_ treatment groups were set, respectively. The cell proliferation activity of WPMY-1 and PrPF cells was determined by CCK-8 assay. The IC50 value was 431.9 *μ*M and 409.2 *μ*M, respectively. The mRNA expression of (b) SOD1 and (c) PAGE4 in WPMY-1 and PrPF cells, which were treated with 400 *μ*M H_2_O_2_ combined with or without 5 mM NAC. (d) Immunoblot assays demonstrated the protein expression of SOD1 and PAGE4 in WPMY-1 and PrPF cells, which were treated with 400 *μ*M H_2_O_2_ combined with or without 5 mM NAC. (e) Immunofluorescence was used to detect the expression of PAGE4 in WPMY-1 cells without or with 400 *μ*M H_2_O_2_ treatment. Left: DAPI (blue) shows nuclear staining; middle: Cy3 immunofluorescence (red) shows PAGE4 protein; right: merged image. Representative graphs were selected into figure. GAPDH is used as loading control. ns: no significant difference; ^∗∗^: *p* < 0.01; ^∗∗∗^: *p* < 0.001. ANOVA or Student's *t*-test. The scale bars are 100 *μ*m.

In order to explore the appropriate concentration of H_2_O_2_, WPMY-1 and PrPF cells were treated with different concentrations of H_2_O_2_ for 8 h and the proliferation activity of the cells was determined. As shown in Figure 2(a), the proliferation activity of WPMY-1 and PrPF cells gradually decreased with the increase of H_2_O_2_ concentration. IC50 of WPMY-1 and PrPF cells were 431.9 *μ*M and 409.2 *μ*M, respectively. Thus, the dose of 400 *μ*M H_2_O_2_ was used to establish the oxidative damage cell model. Furthermore, ROS-related gene superoxide dismutase 1 (SOD1) expression was decreased after H_2_O_2_ treatment in WPMY-1 and PrPF cells, suggesting that prostate stromal cells were indeed experiencing a high level of oxidative stress, and at the same time, NAC that is a penetrating antioxidant significantly reduced the oxidative stress caused by H_2_O_2_ in cells (Figure 2(b)). On the other hand, the mRNA level of PAGE4 was increased over eightfold in cells after H_2_O_2_ treatment (Figure 2(c)), while NAC treatment effectively inhibited the effect of H_2_O_2_ on PAGE4 expression. And the same effect can be seen on the protein levels of SOD1 and PAGE4 (Figure 2(d)). Immunofluorescence demonstrated that there is basically no expression of PAGE4 in WPMY-1 and PrPF cells (Figure 2(e)). However, after H_2_O_2_ treatment, PAGE4 staining was clearly present in prostate stromal cells.

### 3.3. The Downregulation of PAGE4 Inhibits the Cell Survival of Prostate Stromal Cells and Enhances ROS Accumulation under OS

**Figure 3:**
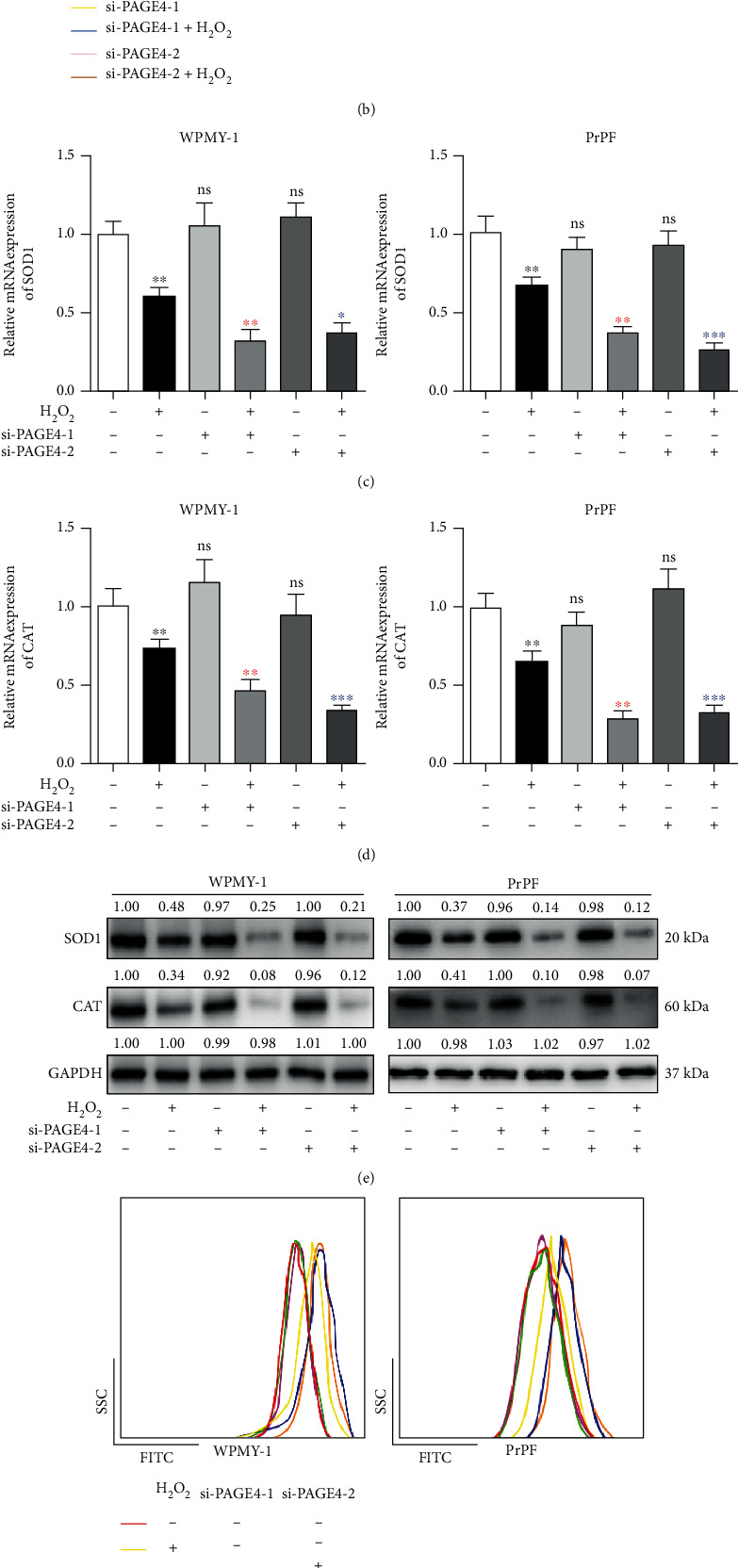
Effect of PAGE4 knockdown on cell proliferation and oxidative stress level of WPMY-1 and PrPF cells under OS. (a) qRT-PCR was used to verify the knockdown efficiency of different siRNAs on PAGE4 in WPMY-1 and PrPF cells at the transcriptional level. (b) The cell viability of WPMY-1 and PrPF after knockdown of PAGE4 with or without 400 *μ*M H_2_O_2_ treatment at different time points by CCK-8 assay. qRT-PCR was used to verify the effect of combined or noncombined 400 *μ*M H_2_O_2_ on the transcription level of (c) SOD1 and (d) CAT after knockdown of PAGE4 in WPMY-1 and PrPF cells. (e) Immunoblot assay shows the effects of PAGE4 knockdown with or without 400 *μ*M H_2_O_2_ on the protein expression levels of SOD1 and CAT in WPMY-1 and PrPF cells. (f) DCFH-DA fluorescent probe was used to analyze the accumulation of ROS by PAGE4 knockdown in WPMY-1 and PrPF cells under OS, and different color curves were used to represent different treatment groups. (g) Statistical analysis of mean fluorescence intensity (MFI) of DCFH-DA in WPMY-1 and PrPF cells after different treatments. GAPDH is used as loading control. ns: no significant difference. In (b), (c), (d), and (g), ^∗^: si-con vs. si-con + H_2_O_2_; red asterisk: si-con + H_2_O_2_ vs. si-PAGE4-1 + H_2_O_2_; blue asterisk: si-con + H_2_O_2_ vs. si-PAGE4-2 + H_2_O_2_; ^∗^: *p* < 0.05; ^∗∗^: *p* < 0.01; ^∗∗∗^: *p* < 0.001. ANOVA or Student's *t*-test.

Additionally, three different PAGE4-specific siRNAs (si-PAGE4s) were transfected into WPMY-1 and PrPF cells to establish the PAGE4-deficient cell model. After 48 h, the mRNA expression of PAGE4 was downregulated by 80%, 77%, and 66% for WPMY-1, while 79%, 80%, and 32% for PrPF, respectively (Figure 3(a)). Therefore, si-PAGE4-1 and si-PAGE4-2 were selected for subsequent experiments, because they exhibited more than 75% inhibitory effect. Cell proliferation was further analyzed by CCK-8 assay for these transfected WPMY-1 and PrPF cells, which revealed that knockdown of PAGE4 significantly inhibited cell proliferation under OS. However, the downregulation of PAGE4 expression alone had no significant effect on cell proliferation (Figure 3(b)). In the presence of H_2_O_2_, the mRNA and protein levels of SOD1 and catalase (CAT) related to oxidative stress were significantly decreased (Figures 3(c)–3(e)). Parallelly, the accumulation of intracellular reactive oxygen species (ROS) was clearly elevated in aforementioned groups (Figures 3(f) and 3(g); Figure S1). However, silencing of PAGE4 alone had no effect on SOD1, CAT, and ROS.

### 3.4. The Downregulation of PAGE4 Intensified OS-Induced Cell Apoptosis, GO/G1 Phase Arrest, and Activation of the JNK Pathway, but Reduced OS-Induced Activation of the ERK Pathway

**Figure 4:**
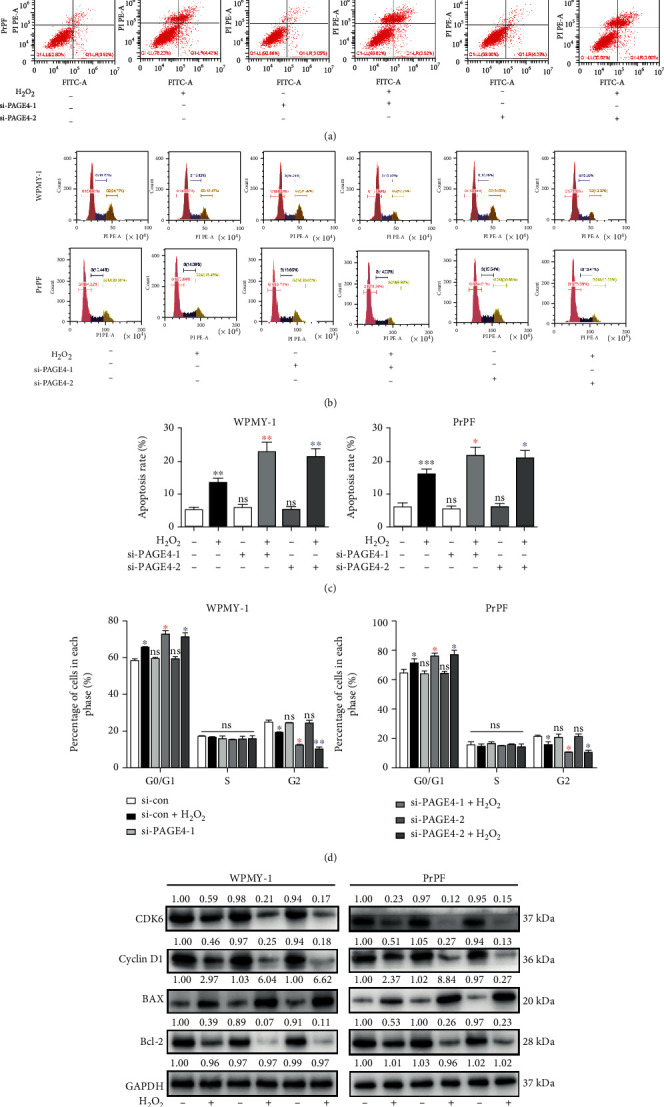
The effects of knockdown of PAGE4 on apoptosis and cell cycle in WPMY-1 and PrPF cells under OS. (a) Flow cytometry was used to detect the effect of combined or not combined 400 *μ*M H_2_O_2_ and knockdown of PAGE4 on the apoptosis level of WPMY-1 and PrPF cells. The lower right quadrant is early apoptosis, and the upper right quadrant is late apoptosis. (b) The effect of PAGE4 knockdown with or without 400 *μ*M H_2_O_2_ on the cell cycle of WPMY-1 and PrPF cells was detected by flow cytometry. Percentage of cell populations at different stages of the cell cycle is listed in the panel (%). (c) The apoptosis rate of each group was statistically analyzed, and the calculated area of apoptosis rate was the Annexin V+/PI+ cell percentage, which was the sum of the percentage of cells in the lower right quadrant and the upper right quadrant. (d) Histogram showing percentage of cell populations at different stages of the cell cycle (%). (e) Immunoblot assays indicating the effects of PAGE4 knockdown with or without 400 *μ*M H_2_O_2_ on the protein expression levels of CDK6, Cyclin D1, BAX, and Bcl-2 in WPMY-1 and PrPF cells. GAPDH is used as loading control. ns: no significant difference; ^∗^: si-con vs. si-con + H_2_O_2_; red asterisk: si-con + H_2_O_2_ vs. si-PAGE4-1 + H_2_O_2_; blue asterisk: si-con + H_2_O_2_ vs. si-PAGE4-2 + H_2_O_2_; ^∗^: *p* < 0.05; ^∗∗^: *p* < 0.01; ^∗∗∗^: *p* < 0.001. ANOVA or Student's *t*-test.

**Figure 5:**
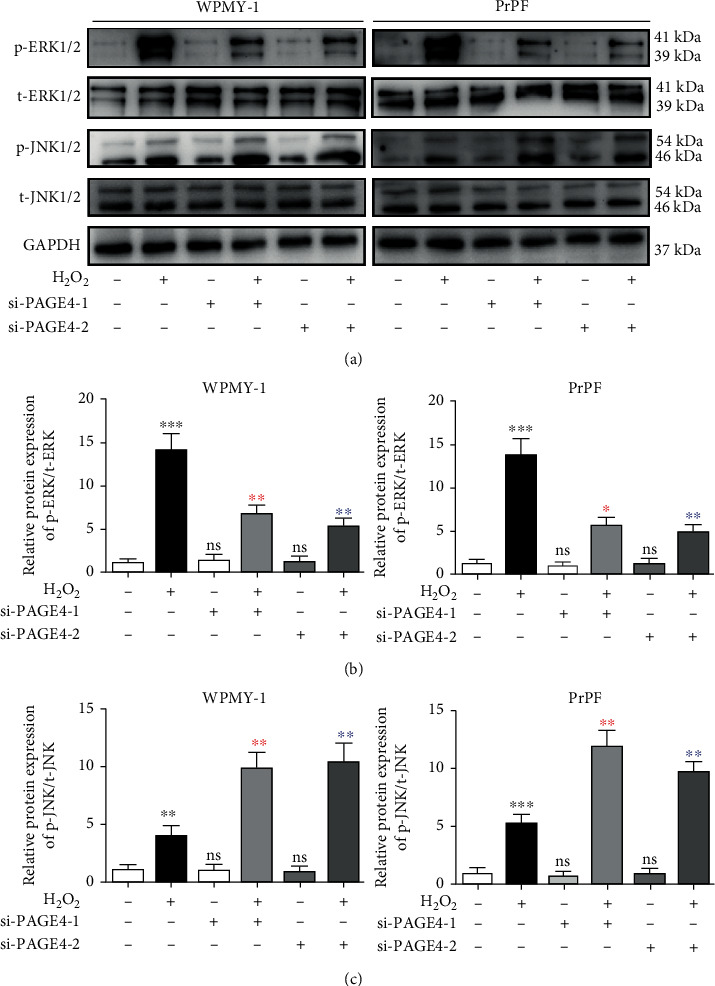
The effects of knockdown of PAGE4 on MAPK signaling pathways of WPMY-1 and PrPF cells under OS. (a) Immunoblot assays showing the effects of PAGE4 knockdown with or without 400 *μ*M H_2_O_2_ on the protein expression levels of p-ERK1/2, t-ERK1/2, p-JNK1/2, and t-JNK1/2 in WPMY-1 and PrPF cells. (b) Relative protein expression abundance of p-ERK/t-ERK in different treatment groups. (c) Relative protein expression abundance of p-JNK/t-JNK in different treatment groups. GAPDH is used as loading control. ns: no significant difference; ^∗^: si-con vs. si-con + H_2_O_2_; red asterisk: si-con + H_2_O_2_ vs. si-PAGE4-1 + H_2_O_2_; blue asterisk: si-con + H_2_O_2_ vs. si-PAGE4-2 + H_2_O_2_; ^∗^: *p* < 0.05; ^∗∗^: *p* < 0.01; ^∗∗∗^: *p* < 0.001. ANOVA or Student's *t*-test.

To better understand the underlying mechanism of this cell survival-inhibiting effect, cell cycle stage and cell apoptosis were further examined via flow cytometry analysis. Compared with the unstimulated cells, H_2_O_2_ stimulation increased apoptosis by more than 2-fold, which was increased even further by additional knockdown of PAGE4 (Figures 4(a) and 4(c)). Similarly, when cells were arrested in the GO/G1 phase by H_2_O_2_ treatment, there was a corresponding shortening of the G2 phase, while there was no significant change in the S phase, and an increase of G0/G1 phase cells approximately 7.3%/6.5% and a decrease of G2 phase cells about 6.4%/5.3% in WPMY-1/PrPF were found. Moreover, the knockdown of PAGE4 not only enhanced the increase of H_2_O_2_-induced GO/G1 phase cells by about 6.6%/5.5% but also enhanced the decrease of H_2_O_2_-induced G2 phase cells by about 6.2%/5.4% in WPMY-1/PrPF (Figures 4(b) and 4(d)). However, siPAGE4 alone demonstrated no effect on cell apoptosis and cell cycle. Accordingly, significant changes were observed for cell apoptosis-associated proteins (BAX and Bcl-2) and cell cycle-related proteins (Cyclin D1 and CDK6) (Figure 4(e)). MAPK signaling pathways are known to regulate cell processes in various ways, including cell proliferation, differentiation, mitosis, and apoptosis. The phosphorylation levels of JNK1/2 and ERK1/2 in MAPK signaling pathways were determined via Western blot. The expression of phosphorylated ERK1/2 (p-ERK1/2) and phosphorylated JNK1/2 (p-JNK1/2) was increased in the cells exposed to H_2_O_2_. The downregulation of PAGE4 attenuated H_2_O_2_-induced p-ERK1/2 activation, but enhanced p-JNK1/2 activation under OS (Figure 5).

### 3.5. The Overexpression of PAGE4 Promotes the Cell Survival of Prostate Stromal Cells and Reduces ROS Accumulation under OS

**Figure 6:**
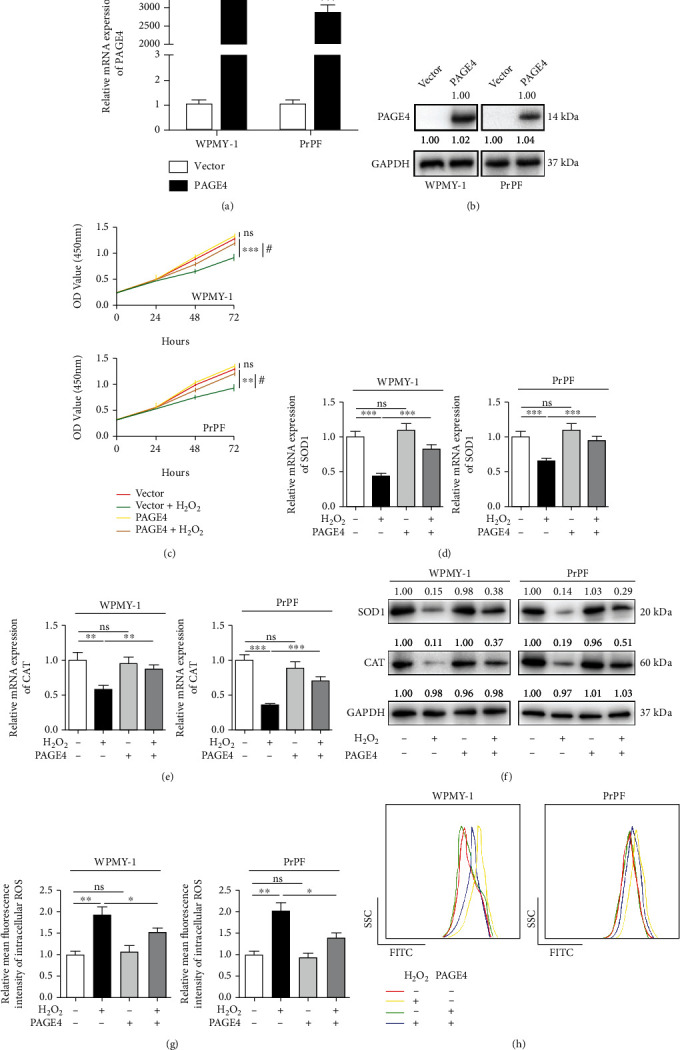
The effect of overexpression of PAGE4 on cell proliferation and oxidative stress level of WPMY-1 and PrPF cells under OS. (a) qRT-PCR was used to verify the efficiency of PAGE4 overexpression in WPMY-1 and PrPF cells at the transcriptional level. (b) Immunoblot assays showing the efficiency of PAGE4 overexpression in WPMY-1 and PrPF cells at the protein level. (c) The cell viability of WPMY-1 (i) and PrPF (ii) after overexpression of PAGE4 with or without 400 *μ*M H_2_O_2_ treatment at different time points by CCK-8 assay (^∗^: vector vs. vector + H_2_O_2_; ^#^: vector + H_2_O_2_ vs. PAGE4 + H_2_O_2_). qRT-PCR was used to verify the effect of PAGE4 and combined or noncombined 400 *μ*M H_2_O_2_ on the transcription level of (d) SOD1 and (e) CAT in WPMY-1 and PrPF cells after PAGE4 overexpression. (f) Immunoblot assays showing the effects of PAGE4 overexpression with or without 400 *μ*M H_2_O_2_ on the protein expression levels of SOD1 and CAT in WPMY-1 and PrPF cells. (g) Statistical analysis of mean fluorescence intensity (MFI) of DCFH-DA in WPMY-1 and PrPF cells after different treatments. (h) DCFH-DA fluorescent probe was used to analyze the accumulation of ROS by PAGE4 overexpression in WPMY-1 and PrPF cells under OS, and different color curves were used to represent different treatment groups. GAPDH is used as loading control. ns: no significant difference; ^∗^: *p* < 0.05; ^∗∗^: *p* < 0.01; ^∗∗∗^: *p* < 0.001. ANOVA or Student's *t*-test.

Additionally, we transfected the plasmid into WPMY-1 and PrPF cells to overexpress PAGE4. qRT-PCR revealed that the mRNA expression of PAGE4 was upregulated by about 3200/2825-fold in WPMY-1/PrPF (Figure 6(a)). The protein level of PAGE4 was obviously increased (Figure 6(b)). Overexpression of PAGE4 could slow down the decline of cell proliferation activity caused by H_2_O_2_-induced acute OS, while it alone had no significant effect on cell proliferation (Figure 6(c)). Moreover, the overexpression of PAGE4 alone exerted no effect on SOD1, CAT, and ROS accumulation, but it ameliorated H_2_O_2_-induced acute OS (Figures 6(d)–6(h), Figure S2).

### 3.6. The Upregulation of PAGE4 Attenuated OS-Induced Cell Apoptosis, GO/G1 Phase Arrest, and Activation of JNK Pathway, but Enhanced OS-Induced Activation of the ERK Pathway

**Figure 7:**
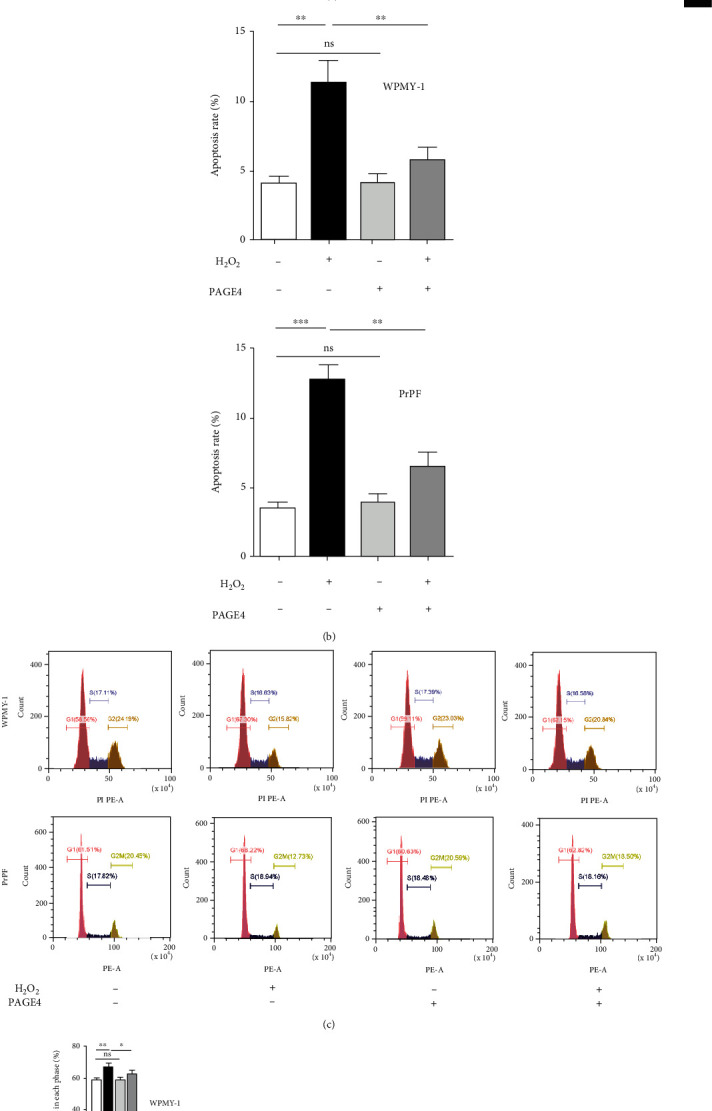
The effects of overexpression of PAGE4 on apoptosis and cell cycle of WPMY-1 and PrPF cells under OS. (a) The effect of PAGE4 overexpression with or without 400 *μ*M H_2_O_2_ on apoptosis levels of WPMY-1 and PrPF cells was detected by flow cytometry. The lower right quadrant is early apoptosis, and the upper right quadrant is late apoptosis. (b) The apoptosis rate of each treatment group was statistically analyzed, and the calculated area of apoptosis rate was the Annexin V+/PI+ cell percentage, which was the sum of the percentage of cells in the lower right quadrant and the upper right quadrant. (c) The effect of PAGE4 overexpression with or without 400 *μ*M H_2_O_2_ on the cell cycle of WPMY-1 and PrPF cells was detected by flow cytometry. Percentages of cell populations at different stages of the cell cycle were listed in the panel (%). (d) Histogram showing percentage of cell populations at different stages of the cell cycle (%). (e) Immunoblot assays showing the effects of PAGE4 overexpression with or without 400 *μ*M H_2_O_2_ on the protein expression levels of CDK6, Cyclin D1, BAX, and Bcl-2 in WPMY-1 and PrPF cells. GAPDH is used as loading control. ns: no significant difference; ^∗^: *p* < 0.05; ^∗∗^: *p* < 0.01; ^∗∗∗^: *p* < 0.001. ANOVA or Student's *t*-test.

**Figure 8:**
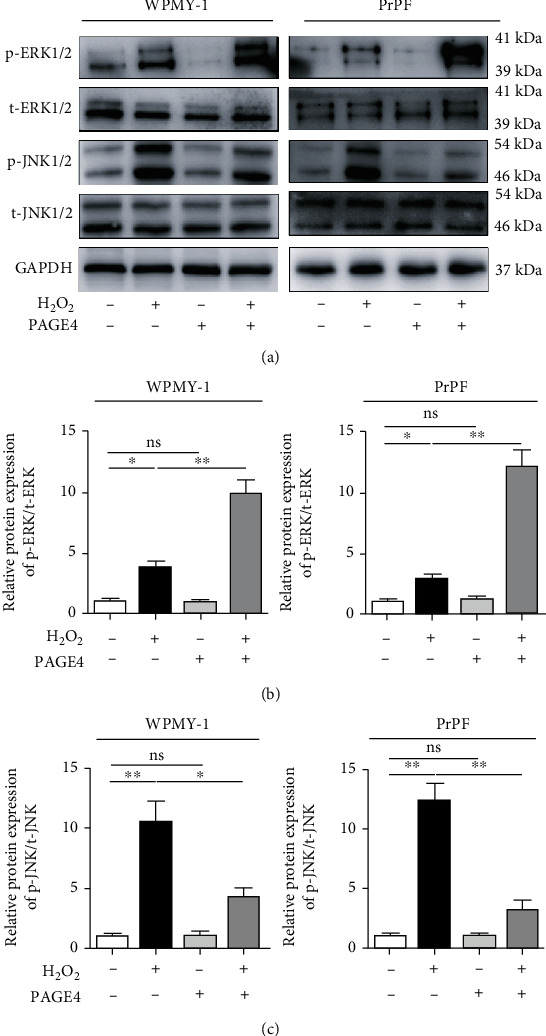
The effects of overexpression of PAGE4 on MAPK signaling pathways of WPMY-1 and PrPF cells under OS. (a) Immunoblot assays showing the effects of PAGE4 overexpression with or without 400 *μ*M H_2_O_2_ on the protein expression levels of p-ERK1/2, t-ERK1/2, p-JNK1/2, and t-JNK1/2 in WPMY-1 and PrPF cells. (b) Relative protein expression abundance of p-ERK/t-ERK in different treatment groups. (c) Relative protein expression abundance of p-JNK/t-JNK in different treatment groups. GAPDH is used as loading control. ns: no significant difference; ^∗^: *p* < 0.05; ^∗∗^: *p* < 0.01. ANOVA or Student's *t*-test.

Similar to PAGE4 knockdown, PAGE4 overexpression alone exhibited no effect on cell apoptosis and cell cycle but it reversed the H_2_O_2_-induced increase in apoptosis, GO/G1 phase arrest, and the corresponding G2 phase shortening (Figures 7(a)–7(d)), as well as related proteins (BAX, Bcl-2, Cyclin D1, and CDK6) (Figure 7(e)). Finally, Western blot revealed overexpression of PAGE4 could potentiate the increase of p-ERK1/2 and offset the increase of p-JNK1/2 triggered by H_2_O_2_ (Figure 8).

## 4. Discussion

The current study determined that PAGE4 was upregulated in hyperplastic prostate and it was mainly localized in the stromal compartment. We further demonstrated that the augmented PAGE4 could promote cell survival by activating the phosphorylation of ERK1/2 and decrease cell apoptosis by inhibiting the phosphorylation of JNK1/2 under the presence of OS, which could contribute to the development of BPH.

Although PAGE4 is a prostate-specific protein, it is highly dynamic. It is richly expressed in the prostate of the fetus at 21 weeks of gestational age, but almost undetectable after 36 weeks [15], and can be reupregulated in some benign and malignant prostate diseases. The current study demonstrated the richly expressed PAGE4 in BPH tissue both at mRNA and protein levels, while it was barely detectable in normal adult prostate. Of interest, it was predominantly localized in the stroma. Previous studies have shown that PAGE4 was located in mitochondria and that the expression level of PAGE4 was related to OS. In the aged prostate, cells are often subjected to severe OS due to obesity [23], smoking [24, 25], and aging [26]. It has been reported that the level of OS markers was significantly increased in the plasma [27, 28] and urine [29] specimens of patients with BPH. Thus, OS could contribute to BPH via modulating the PAGE4 pathway.

In order to simulate the environment of oxidative stress, we created an oxidative stress cell model induced by 400 *μ*M H_2_O_2_, which was near its IC50 (IC50_WPMY-1_: 431.9 *μ*M, IC50_PrPF_: 409.2 *μ*M). Thus, the apoptosis and inhibition of cell proliferation induced by H_2_O_2_ were independent of cytotoxic effects. With H_2_O_2_ stimulation, the expression of PAGE4 in WPMY-1 cells and PrPF was significantly increased at both the transcription and translation levels. Moreover, inhibited cell proliferation, enhanced cell apoptosis, and the arrest of G0/G1 phase of cells were observed under the condition of acute OS. At the basic level, OS is crucial for cells to perform normal biological functions and signal transduction [30]. When ROS production is not coordinated with the biological system to repair oxidative damage, the direct effect of excessive ROS can inhibit cell proliferation and promote cell apoptosis via oxidative killing [31]. However, knockdown or overexpression of PAGE4 alone had no effect on cell growth and cell cycle as well as intracellular OS markers. Interestingly, H_2_O_2_ combined with PAGE4 silencing potentiated intracellular OS, cell apoptosis, along with arrest of cell cycle, and inhibited cell proliferation. Parallelly, H_2_O_2_ combined with PAGE4 overexpression ameliorate the aforementioned process. Therefore, our study indicates that PAGE4 was upregulated in the presence of OS which lead to reduction of intracellular OS levels and promotion of cell survival. When the level of oxidative stress in the environment reached a certain threshold, PAGE4 was activated as a stress response protein to reduce OS-induced damage [17].

We further explored the possible mechanisms of OS-PAGE4 on the development of BPH. It is well known that MAPK signaling pathways regulate cell physiology via various pathways, including cell proliferation, differentiation, mitosis, cell survival, and apoptosis [32, 33]. Indeed, our previous studies have shown that the MAPK signaling pathway is related to the development of BPH [13]. It is also been demonstrated that the activation of JNK1/2, a downstream gene of the MAPK pathway [34, 35], can increase apoptosis. Additionally, another MAPK family member ERK1/2 participates in cell cycle regulation [36, 37]. In the present study, the MAPK/JNK pathway and the MAPK/ERK pathway were activated after H_2_O_2_ treatment. Meanwhile, H_2_O_2_ combined with silencing PAGE4 enhanced the activation of JNK1/2 and weakened the activation of ERK1/2. On the contrary, H_2_O_2_ combined with overexpression of PAGE4 weakened the activation of JNK1/2 and enhanced the activation of ERK1/2. It is speculated that oxidative stress induces cell death partly through activating the JNK pathway which has been known to be able to trigger an apoptotic cascade [38, 39]. In contrast, the ERK pathway that promotes cell survival is also activated under OS, acting in conjunction with the JNK pathway to balance cell death [40]. In this context, PAGE4 protect cells from oxidative stress potentially by reducing cell death while enhancing cell survival. Lv et al. [17] also demonstrated the large number of colocalization sites of activated ERK1/2 and PAGE4 in human prostate tissues [41]. Considering that PAGE4 does not exist as a kinase, its activation of the MAPK signaling pathway would be indirect. OS was not only a direct activator of the JNK pathway [42, 43] but also an indirect activator of the ERK pathway through vascular endothelial growth factor [44]. In addition, there was abundant signal cross-talk between the JNK and ERK pathways, and it was likely that the cross-activation of ERK and JNK contributed to the induction of cell division or eventual differentiation [45]. Therefore, long-term chronic oxidative stress, as an intermediary between PAGE4 and the MAPK signaling pathway, produced indirect and direct activation effects on ERK and JNK pathways, respectively. In addition, SOD1 and CAT, as antidotes of ROS, had antagonistic effects against OS. Actually, ROS levels were in dynamic balance with antioxidants such as SOD1 and CAT. When ROS levels were abnormally elevated, this balance was disrupted, resulting in decreased SOD1 and CAT expression, increased ROS accumulation, and activation of ERK and JNK pathways. On the other hand, the anti-OS function of PAGE4 needed to be activated in the presence of OS, upregulating SOD1 and CAT, thereby reducing ROS levels, activating p-ERK1/2, and inhibiting p-JNK1/2 in the regulation of the ERK-JNK interactive network. Finally, PAGE4 inhibited cell apoptosis and promoted cell proliferation. As one of the contributing factors of BPH, PAGE4 exists in the complex pathogenesis of BPH and participates in the occurrence and development of BPH.

Histopathologically, BPH is characterized by nodular hyperplasia of epithelial and stromal components, and the stromal-epithelial interaction mediated by paracrine signals between the epithelium and stromal is an important factor. Prostatic stromal cells can secrete growth factors (such as fibroblast growth factor and insulin-like growth factor I and II) and other cytokines (IL-2, IL-4, and IFN-*γ*) to regulate the proliferation and differentiation of epithelial cells and stroma itself [46, 47]. As an abnormally expressed gene in the stroma of hyperplastic prostate, PAGE4 promotes the survival of stromal cells under OS, and the latter may affect the epithelial cell proliferation of BPH through stromal-epithelial interaction. Its specific mechanism needs to be further studied.

The limitation of the current study is using the acute OS cell model used to mimic the chronic OS occurring in the hyperplastic prostate. At present, there is no suitable chronic OS cell model for prostate cell signaling.

## 5. Conclusion

Our study demonstrates that PAGE4 enhances the survival of prostate stromal cells under oxidative stress. PAGE4 was activated via the stimulation of ROS production, promoting cell survival and reducing cell apoptosis by increasing the phosphorylation of ERK1/2 and decreasing the phosphorylation of JNK1/2, finally leading to the development and progression of BPH. Therefore, the high expression of PAGE4 in the pathologic prostate can be a potential novel therapeutic target for BPH.

## Figures and Tables

**Table 1:** List of primers for qRT-PCR.

Gene	Symbol	Forward primer (5′-3′)	Reverse primer (5′-3′)	Annealing temperature (°C)	Length (bp)
Prostate-associated gene 4	PAGE4	5′-TGAGGTCTAAACAAGGAGGCAG-3′	5′-CACGCTCACTCCGAGTCTTT-3′	60	152
Superoxide dismutase 1	SOD1	5′-TGTGGCCGATGTGTCTATTG-3′	5′-GCGTTTCCTGTCTTTGTACTTTC-3′	60	124
Catalase	CAT	5′-CAGATAGCCTTCGACCCAAG-3′	5′-GTAGGGACAGTTCACAGGTATATG-3′	60	96
Glyceraldehyde-3-phosphate dehydrogenase	GAPDH	5′-TGCACCACCAACTGCTTAG-3′	5′-GATGCAGGGATGATGTTC-3′	60	176

**Table 2:** List of primary antibodies.

Antigens	Species antibodies raised in	Dilution used	Supplier
PAGE4, human	Rabbit, monoclonal	1 : 1000 (WB)	Thermo Fisher Scientific, USA, Cat. #PA5-42147
PAGE4, human	Rabbit, monoclonal	1 : 200 (IF)	Abcam, UK, Cat. #ab224454
SOD1, human	Rabbit, monoclonal	1 : 1000 (WB)	Abcam, UK, Cat. #ab51254
CAT, human	Rabbit, monoclonal	1 : 1000 (WB)	Abcam, UK, Cat. #ab112799
CDK6, human	Rabbit, monoclonal	1 : 1000 (WB)	Cell Signaling Technology, USA, Cat. #13331
Cyclin D1, human	Rabbit, monoclonal	1 : 1000 (WB)	Cell Signaling Technology, USA, Cat. #2978
BAX, human	Rabbit, monoclonal	1 : 1000 (WB)	Cell Signaling Technology, USA, Cat. #5023
Bcl-2, human	Rabbit, monoclonal	1 : 1000 (WB)	Cell Signaling Technology, USA, Cat. #2872
ERK1/2, human	Rabbit, monoclonal	1 : 1000 (WB)	ABclonal, USA, Cat. #A10813
Phospho-ERK1/2, human	Rabbit, monoclonal	1 : 1000 (WB)	ABclonal, USA, Cat. #AP0427
JNK1/2, human	Rabbit, monoclonal	1 : 1000 (WB)	ABclonal, USA, Cat. #A11119
Phospho-JNK1/2, human	Rabbit, monoclonal	1 : 1000 (WB)	Cell Signaling Technology, USA, Cat. #4668
GAPDH, human	Rabbit, polyclonal	1 : 1000 (WB)	ABclonal, USA, Cat. #AC027
*α*-SMA, human	Mouse, monoclonal	1 : 200 (IF)	Santa Cruz, sc-53142

**Table 3:** List of secondary antibodies.

Secondary detection system used	Host	Dilution used	Supplier
Anti-mouse-IgG (H+L)-HRP	Goat	1 : 10,000 (WB)	Sungene Biotech, China, Cat. #LK2003
Anti-rabbit-IgG (H+L)-HRP	Goat	1 : 10,000 (WB)	Sungene Biotech, China, Cat. #LK2001
Anti-rabbit IgG (H+L), F(ab′)2 fragment (Alexa Fluor®488 conjugate)	Goat	1 : 50 (IF)	Cell Signaling Technology, Cat. #4412
Hoechst 33342 (1 mg/ml) nucleic acid staining (DAPI) fragment (Alexa Fluor® 488)	—	1 : 750 (IF)	Molecular Probes/Invitrogen, Carlsbad, CA, USA, Cat. no. A11007

## Data Availability

The data used to support the findings of this study are available from the corresponding author upon reasonable request.

## Supplementary Materials

Supplementary Materials Figure S1: effect of PAGE4 knockdown on ROS accumulation in WPMY-1 and PrPF cells under OS. A: DCFH-DA fluorescent probe was used to analyze the accumulation of ROS by PAGE4 knockdown in WPMY-1 cells under OS. B: DCFH-DA fluorescent probe was used to analyze the accumulation of ROS by PAGE4 knockdown in PrPF cells under OS. Figure S2: effect of PAGE4 overexpression on ROS accumulation in WPMY-1 and PrPF cells under OS. A: DCFH-DA fluorescent probe was used to analyze the accumulation of ROS by PAGE4 overexpression in WPMY-1 cells under OS. B: DCFH-DA fluorescent probe was used to analyze the accumulation of ROS by PAGE4 overexpression in PrPF cells under OS.
