# Supplementary material for: The Prostate-Associated Gene 4 (PAGE4) Could Play a Role in the Development of Benign Prostatic Hyperplasia under Oxidative Stress
Source: Oxid Med Cell Longev. 2022 May 19;2022:7041739. doi: 10.1155/2022/7041739 (PMC9135540; doi:10.1155/2022/7041739)
Supplement: Supplementary Materials — Figure S1: effect of PAGE4 knockdown on ROS accumulation in WPMY-1 and PrPF cells under OS. A: DCFH-DA fluorescent probe was used to analyze the accumulation of ROS by PAGE4 knockdown in WPMY-1 cells under OS. B: DCFH-DA fluorescent probe was used to analyze the accumulation of ROS by PAGE4 knockdown in PrPF cells under OS. Figure S2: effect of PAGE4 overexpression on ROS accumulation in WPMY-1 and PrPF cells under OS. A: DCFH-DA fluorescent probe was used to analyze the accumulation of ROS by PAGE4 overexpression in WPMY-1 cells under OS. B: DCFH-DA fluorescent probe was used to analyze the accumulation of ROS by PAGE4 overexpression in PrPF cells under OS. [file 7041739.f1.docx]

**Fig. S1 Effect of PAGE4 knockdown on ROS accumulation in WPMY-1 and PrPF cells under OS.**


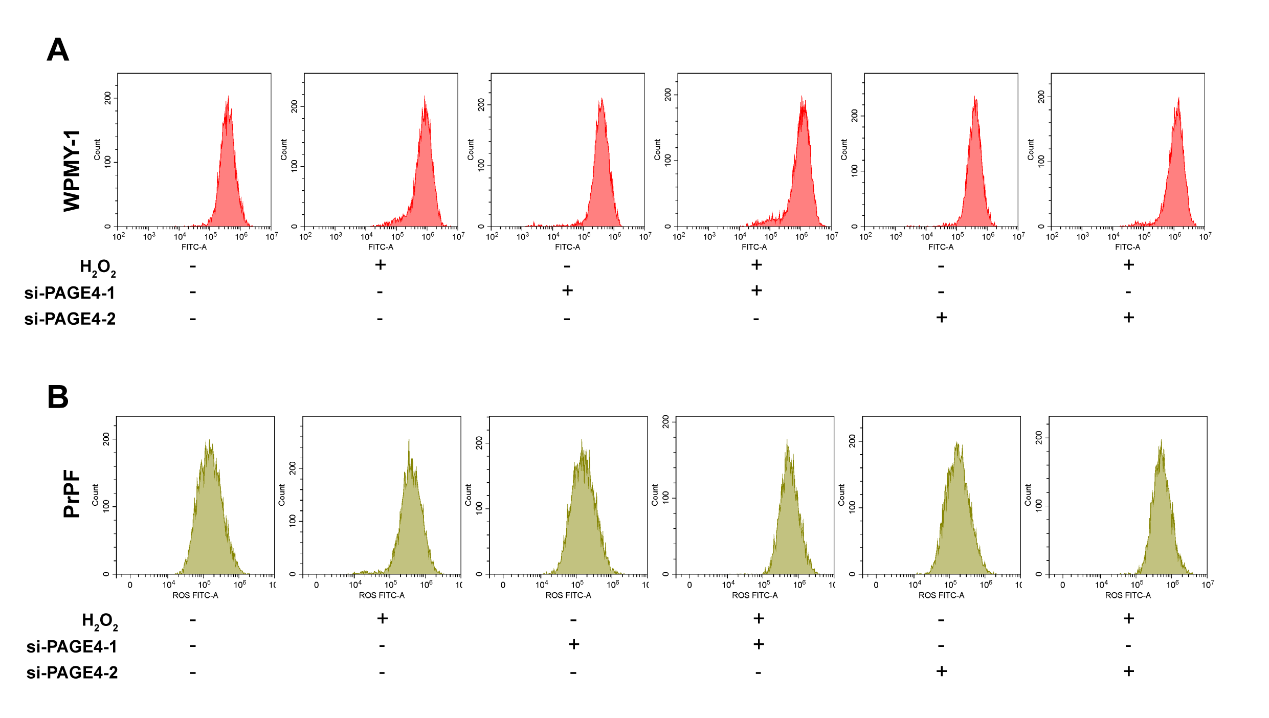


**A:** DCFH-DA fluorescent probe was used to analyze the accumulation of ROS by PAGE4 knockdown in WPMY-1 cells under OS. **B:** DCFH-DA fluorescent probe was used to analyze the accumulation of ROS by PAGE4 knockdown in PrPF cells under OS.

**Fig. S2 Effect of PAGE4 overexpression on ROS accumulation in WPMY-1 and PrPF cells under OS.**


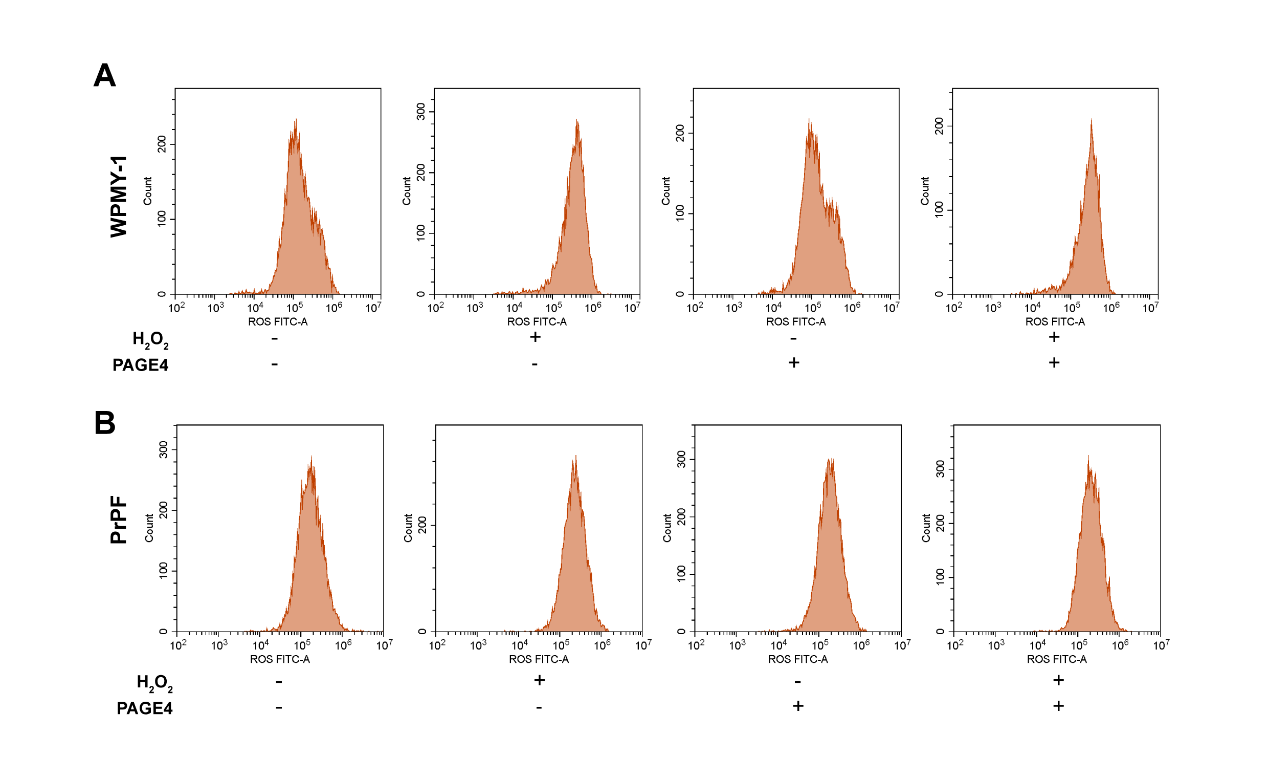


**A:** DCFH-DA fluorescent probe was used to analyze the accumulation of ROS by PAGE4 overexpression in WPMY-1 cells under OS. **B:** DCFH-DA fluorescent probe was used to analyze the accumulation of ROS by PAGE4 overexpression in PrPF cells under OS.
